# Obstetrics and Gynecology Provider Experience Screening for Harmful Alcohol Use: An Unmet Need for Standardized Screening and Intervention

**DOI:** 10.7759/cureus.72795

**Published:** 2024-10-31

**Authors:** Mary J Thomson, Cresta Jones, Nicholas Lim

**Affiliations:** 1 Gastroenterology, Hepatology and Nutrition, University of Minnesota, Minneapolis, USA; 2 Obstetrics, Gynecology and Women's Health, University of Minnesota, Minneapolis, USA

**Keywords:** addiction medicine, alcohol-related liver disease, alcohol use disorder, multidisciplinary care, women’s health

## Abstract

Introduction

Early identification of at-risk alcohol use is key to preventing complications of alcohol use disorder (AUD). Obstetrics and gynecology (OB/GYN) providers are in a unique position to screen for alcohol use in young female patients who may not otherwise seek traditional primary care. The purpose of this study was to learn how OB/GYN providers screen for and manage harmful alcohol use in their clinical care.

Method

We surveyed OB/GYN providers in a single health care system regarding how they screen for and manage alcohol use. The results were reported using descriptive statistics. Bivariate analysis to assess the impact of physician characteristics on responses was performed using chi-square testing.

Results

From the results, it was observed that 64.7% and 60.6% of OB/GYN providers screen for alcohol use most or all of the time during obstetrics and gynecologic visits, respectively. Less than half of providers refer patients with at-risk use for further interventions or treatment (41.0% referred to an addiction specialist, 41.0% recommended discussing their behavior with their PCP, 35.9% referred to social work, 10.4% referred to gastroenterology or hepatology). Providers who screened did so most commonly by directly asking patients about their alcohol use (70.6% during obstetrics and 60.1% during gynecologic visits). Only 11.8% and 15.2% of providers who screened did so using standardized screening methods (e.g. CAGE (Cut down, Annoyed, Guilty, and Eye-opener) questionnaire and Alcohol Use Disorders Identification Test-Consumption (AUDIT-C)) during obstetrics and gynecologic visits, respectively. Only 30.8% of the providers felt they received adequate training on AUD. Providers who had experience taking care of a patient with severe alcohol-related liver disease (100% v. 0%, χ^2^=4.69, p=0.047) or died from this (100% v. 0%, χ^2^=11.35, p<0.01) were more likely to refer to gastroenterology.

Conclusions

Further work needs to be done to improve screening for and management of harmful alcohol use in OB/GYN clinics. Standardized screening methods were rarely used. Education of OB/GYN providers on AUD management and facilitating referrals for addiction care are opportunities to prevent the consequences of AUD in at-risk female patients.

## Introduction

Alcohol use disorder (AUD) is becoming a major public health concern for women. While men have historically consumed more alcohol, evidence shows the gender gap is narrowing and the rates of alcohol use are rising more in women [1]. Women of childbearing age make up much of this trend [2]. Alcohol use during pregnancy is associated with adverse outcomes including higher rates of miscarriage and fetal alcohol syndrome [3]. Mirroring the rise in alcohol use, there has been a surge in alcohol-related liver disease (ALD). Women have had a disproportionately higher increase in the incidence of alcohol-related hepatitis [4] and die from ALD earlier than men [5]. This is partly due to alcohol being more hepatotoxic in women due to altered pharmacokinetics and sex-related hormonal differences [6, 7].

Early identification of and intervention for AUD is key to the prevention of downstream medical complications. However, younger patients do not often see a health care provider who could assess their alcohol use: 44% of adults aged 20-29 years do not have a primary care provider [8]. However, women without chronic conditions are more likely to identify their obstetrics and gynecology (OB/GYN) provider as their primary care provider (PCP) [9]. OB/GYN providers are therefore in a unique position to discuss alcohol use in younger female patients. The aim of this survey was to better understand how OB/GYN providers screen for and manage harmful alcohol use in their practice. This article was previously presented as a meeting abstract at the 2023 AASLD Liver Meeting on November 12, 2023.

## Materials and methods

An anonymous, online-based survey was distributed to OB/GYN providers at the M Health Fairview health system. Ten clinical sites were included, covering the Minneapolis-St. Paul metropolitan and surrounding areas. OB/GYN providers (physicians, certified nurse midwives, nurse practitioners, and physician assistants) were eligible if they participated in outpatient care. This project was approved by the University of Minnesota IRB (STUDY00015405). Informed consent was waived due to the anonymous nature of the survey. Providers were asked how they screen for alcohol use, methods used to do so, typical follow-up once at-risk use was identified, and their experience with AUD and ALD (Supplementary File in the Appendix). Providers were asked about gynecologic and obstetrics visits separately. The results were reported using descriptive statistics. Bivariate analysis to assess the impact of physician characteristics on responses was performed using chi-square testing using Stata (StataCorp LLC, College Station, TX).

## Results

Provider characteristics

Of the 143 OB/GYN providers who received the survey, 27.3% (n=39) completed it. Thirty-four providers reported seeing patients who were pregnant (obstetrics visits) and 33 providers reported seeing patients who were not pregnant (gynecologic visits). The majority of the responders identified as female (94.8%), Caucasian (84.2%) and the median age was 41 years (IQR 34-46). More than half (53.8%) were physicians (Doctor of Medicine (M.D.) or Doctor of Osteopathy (D.O.) degrees), followed by 43.6% with training as a certified nurse midwife. One-third (35.9%) were in training or had completed their medical training within the past five years. More than 90% of providers reported completing health maintenance tasks when indicated, including screening for diabetes, colon cancer, breast cancer, and tobacco use.

Provider experience with alcohol use disorder and alcohol-related liver disease

Over half of the providers (53.9%) thought that harmful alcohol use was a moderate problem in their community. Almost all (97.4%) had taken care of a patient with a history of harmful use, while 48.7% of the responders had cared for a patient with severe ALD (cirrhosis or alcohol-related hepatitis with jaundice) and 28.2% cared for a patient who died from ALD. Under a third (30.8%) felt they had received adequate training on harmful alcohol use during their training and almost all (92.3%) were interested in learning more about AUD and ALD.

Alcohol use screening and management during OB/GYN visits

More than 60% reported screening for harmful alcohol use in both obstetrics and gynecology visits either most or all of the time, and the majority felt somewhat or very comfortable doing so. For providers who counseled patients about the risk of alcohol, half (45.5%) said the upper limit of safe alcohol use in non-pregnant patients was no more than one drink a day, up to four days a week. Almost all (94.1%) did not feel any alcohol use was safe in pregnant patients (Table 1). 

**Table 1:** OB/GYN Provider Survey Results: Screening and Safe Alcohol Use The providers were asked about how they screen for and counsel on safe alcohol during obstetrics and gynecologic visits separately.

	Gynecology Visits (n=33)	Obstetrics Visits (n=34)
How often do you perform standardized screening for alcohol use?		
Never	9.1% (n=3)	2.9% (n=1)
Rarely	15.2% (n=5)	17.7% (n=6)
Sometimes	15.2% (n=5)	14.7% n=5)
Most of the time	42.4% (n=14)	29.4% (n=10)
All of the time	18.2% (n=6)	35.3% (n=12)
How comfortable are you screening for alcohol use?		
Not at all comfortable	0% (n=0)	0% (n=0)
Not very comfortable	18.2% (n=6)	8.8% (n=3)
Somewhat comfortable	42.4% (n=14)	41.2% (n=14)
Very comfortable	39.4% (n=13)	50.0% (n=17)
What do you recommend as the safe upper limit of alcohol use?		
I do not feel any alcohol use is safe	0% (n=0)	94.1% (n=32)
No more than 1 drink a week	0% (n=0)	2.9% (n=1)
No more than 1 drink a day, up to 4 days a week	45.5% (n=15)	2.9% (n=1)
No more than 1 drink a day, up to 7 days a week	27.3% (n=9)	0% (n=0)
No more than 2 drinks a day, up to 4 days a week	6.1% (n=2)	0% (n=0)
No more than 2 drinks a day, up to 7 days a week	3.0% (n=1)	0% (n=0)
I do not have a recommendation	18.2% (n=6)	0% (n=0)

**Figure 1:**
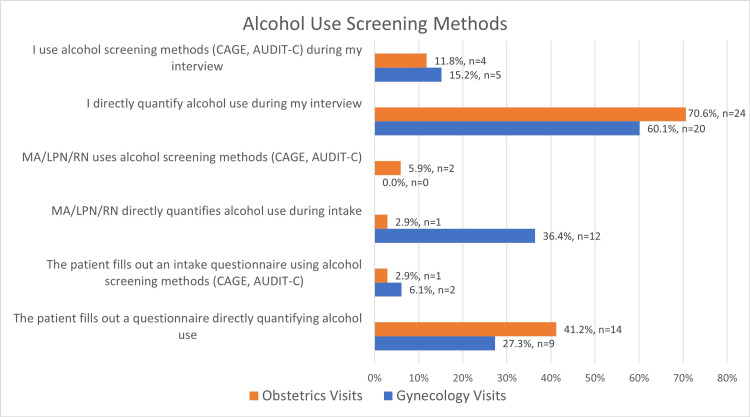
Provider Methods Used Screen for Alcohol Use Providers chose up three methods to describe how they screened for alcohol use during Gynecology and Obstetrics visits. AUDIT-C: Alcohol Use Disorders Identification Test-Consumption CAGE questionnaire: Cut, Annoyed, Guilty, and Eye-opener MA: Medical Assistant LPN: Licensed Practical Nurse RN: Registered Nurse

The providers most commonly screened by asking patients directly about their alcohol use. Screening tools such as CAGE (Cut down, Annoyed, Guilty, and Eye-opener; developed by Johns Hopkins Medicine) questionnaire and Alcohol Use Disorders Identification Test-Consumption (AUDIT-C) were not commonly used (Figure 1).

The provider factors associated with screening for alcohol use most or all of the time were evaluated. Providers who were five or less years out of medical training were less likely to screen for alcohol use during gynecologic visits (27.3% of providers who were five or less years out of training screened for alcohol use v. 77.3% providers who were more than five years out of training, χ2=7.68, P<0.01). Other provider factors were not associated with screening patterns (Table 2). No provider characteristics were associated with screening for at-risk alcohol use during obstetrics visits (Table 2).

**Table 2:** Provider Associations with Screening for Alcohol Use Most or All of the Time during Gynecologic and Obstetrics visits *Pearson's chi-square test (χ2 test) Categories were self-reported by the providers.

		Gynecologic Visits				Obstetrics Visits			
Group A	Group B	Group A % screening most or all of the time	Group B % screening most or all of the time	Chi-square value*	P-value	Group A % screening most or all of the time	Group B % screening most or all of the time	Chi-square value*	P-value
Physician	Non-physician	61.1	60.0	0.00	1.00	52.9	76.5	2.06	0.28
Academic	Non-academic	68.8	52.9	0.86	0.48	56.3	72.2	0.95	0.48
Urban	Non-urban	66.7	50.0	0.89	0.47	57.1	76.9	1.38	0.29
Suburban	Non-suburban	60.0	60.9	0.00	1.00	72.7	60.9	0.46	0.71
≤ 5 years out of training	> 5 years out of training	27.3	77.3	7.68	0.01	58.3	68.2	0.33	0.71

The provider experience with ALD was not associated with screening for alcohol use most or all of the time. During gynecologic visits, 60.0% of those who had taken care of a patient with severe ALD screened for alcohol use compared to 61.1% of those who had not (χ2=0.00, p=1.00). During obstetrics visits, 57.1% of those who had taken care of a patient with severe ALD screened compared to 70.0% who had not (χ2=0.60, p=0.487). Forty percent of providers who took care of someone who died from ALD screened for alcohol use during gynecologic visits compared to 60.0% who had not (χ2=2.26, p=0.245), and 71.4% of providers who took care of someone who had died from ALD screened during obstetrics visits compared to 63.0% who had not (χ2=0.17, p=1.000).

In providers who reported not screening during gynecologic visits, their reasons included the inability to effectively screen (28.6%), counsel (28.6%), or refer (28.6%) patients with harmful alcohol use. Providers who did not screen for at-risk use during obstetrics visits cited time constraints (33.3%), worries about legal consequences for the patient (33.3%), and not feeling able to effectively refer patients with harmful use (33.3%).

Alcohol use counseling

When harmful use was identified, most providers (89.8%) counseled patients on safe use, but less than half referred the patient to further care (41.0% referred to an addiction specialist, 41.0% recommended discussing their behavior with their personal care physician (PCP), 35.9% referred to social work, and 10.4% referred to gastroenterology or hepatology).

Providers who had taken care of a patient who had severe ALD were more likely to refer to gastroenterology or hepatology for further care (100% of providers who had taken care of a patient with severe ALD referred v. 0% who had not taken care of a patient with severe ALD, χ^2^=4.69, p=0.047). Providers who had taken care of someone who died from ALD were also more likely to refer to gastroenterology or hepatology (100% of providers who had taken care of a patient who died of ALD referred v. 0% who had not taken care of a patient who died of ALD, χ^2^=11.35, p=0.004).

## Discussion

The American College of Obstetricians and Gynecologists recommends that OB/GYN providers screen for and intervene in alcohol misuse [10]. In this survey, the majority of OB/GYN providers at a large health system screen their patients for alcohol use, albeit less commonly than other health maintenance tasks such as diabetes screening. OB/GYN providers completing our survey rarely used validated screening methods to identify at-risk use.

Patients who indulged in harmful alcohol use were ultimately found to have AUD and benefited from intensive interventions, including discussion of counseling and medications to assist with cravings [11]. Compared to male patients, female patients with AUD have more barriers and are less likely to access AUD treatment, which are related to differences in income, employment, and care responsibilities for children living at home [12]. In the United States, the Affordable Care Act expanded insurance coverage for many, but low-income female patients in states that did not expand Medicaid eligibility may not have affordable health insurance coverage outside of pregnancy [13]. Thus OB/GYN providers could be key to helping more women access comprehensive treatment for AUD. Less than half of providers in this survey referred their patients for additional care after identifying harmful alcohol use. Provider experience with complications of AUD plays a role, as OB/GYN providers who had taken care of a patient with severe ALD were more likely to refer patients with at-risk alcohol use to gastroenterology or hepatology.

A lack of training was identified as one reason that could result in a lack of self-efficacy in caring for patients with AUD. These results were similar to other studies showing providers lack confidence in screening and intervening in case of harmful alcohol use in women for receiving prenatal care [14] and do not use standardized screening tools often [15]. The American College of Obstetricians and Gynecologists and the National Institute on Alcohol Abuse and Alcoholism created provider educational materials on harmful alcohol use, but a previous survey found most OB/GYN providers were not aware of these resources [16]. At both a resident and provider level, further continuing medical education in addiction medicine, including the use of validated alcohol screening tools, may help to address these issues and improve OB/GYN providers’ use of brief interventions for harmful use [17,18]. In fact, the implementation of a pilot substance use disorder curriculum for OB/GYN residents, which included training on motivational interviewing and brief interventions, was found to improve provider confidence in managing these disorders [19]. Health systems could also develop multidisciplinary care pathways for OB/GYN providers to refer their patients with harmful alcohol use for a formalized alcohol assessment with a licensed alcohol and drug counselor. In other primary care settings, the use of telemedicine or clinic co-localization has shown success in improving access and utilization of integrated care models that link addiction medicine and OB/GYN care [20,21].

The limitations of this study include its small sample size and single health care center location, although multiple clinical sites were used. A strength of our study is the assessment of screening for alcohol use in OB/GYN practice, as previous studies have focused on primary care clinics only [22], or only patients who are pregnant [15]. 

## Conclusions

The majority of OB/GYNs are screening their patients for alcohol use but are not referring them for additional interventions or treatment once at-risk use is identified. As more young women than ever are developing AUD, OB/GYN providers could play an important role in early identification and treatment, possibly preventing future morbidity and mortality in this patient population. Education of OB/GYN providers on the management of AUD and facilitation of referrals to addiction medicine may bridge the current treatment gap that occurs after the identification of AUD.

## Appendices

Supplemental File: Provider Survey

Screening for Alcohol Use at Gynecologic Visits

The following set of questions refers to gynecology clinic visits in a non-pregnant patient.

Screener Question 1: In your practice do you see non-pregnant patients for gynecology visits?

Yes

No

Skip to Header 2 if the answer to the previous question is “No”

Q1 How often do you perform standardized screening for alcohol use during gynecology visits? (This does not include discussing alcohol use after a patient brings up the topic.)

All of the time

Most of the time 

Sometimes

Rare

Never

Q2 How comfortable are you screening for alcohol use during gynecology visits?

Very comfortable

Somewhat comfortable

Not very comfortable

Not at all comfortable

Display the following question if you answered “All of the time”, “Most of the time”, “Sometimes”, or “Rarely” to Q1

Q3 How do you screen for alcohol use during gynecology visits? (Select up to three methods used most often.)

The patient fills out an intake questionnaire directly quantifying alcohol use.

The patient fills out an intake questionnaire using alcohol screening methods (CAGE, AUDIT-C).

An MA/LPN/RN directly quantifies alcohol use during intake.

An MA/LPN/RN uses alcohol screening methods (CAGE, AUDIT-C) during intake.

I directly quantify alcohol use during my interview.

I use alcohol screening methods (CAGE, AUDIT-C) during my interview.

Display the following question if you answered “Never” to Q1

Q4 What are the reasons you do not routinely screen for alcohol use during gynecology visits? (Select up to three of the most common reasons.)

There is not enough time during clinic visits.

Screening for at-risk or harmful alcohol use is outside the scope of OB/GYN and should be done in primary care.

I am worried it will interfere with patient/provider relationship.

I am worried it will lead to legal consequences for the patient.

I do not feel I have the ability to effectively screen for at-risk or harmful alcohol use.

I do not feel I have the ability to effectively counsel patients about at-risk or harmful alcohol use.

I do not feel I have the ability to effectively refer patients with at-risk or harmful alcohol use for treatment.

Q5 Do you ever counsel non-pregnant patients about at-risk or harmful alcohol use?

Yes

No

Q6 Which of the following best describes what you recommend as the upper limit for a safe amount of alcohol in non-pregnant patients?

I do not feel any alcohol use is safe

No more than 1 drink per week

No more than 1 drink per day, Up to 4 days a week

No more than 1 drink per day, Up to 7 days a week

No more than 2 drinks per day, Up to 4 days a week

No more than 2 drinks per day, Up to 7 days a week

I do not have a recommendation

Header 2 Screening for Alcohol Use at Obstetric Visits The following set of questions refer to obstetric visits in a patient who is pregnant or hoping to become pregnant
 

Screener 2 In your practice do you see patients who are pregnant or hoping to become pregnant for obstetric visits?

Yes

No

Skip to Header 3 if the answer to the previous question is “No”

Q7 How often do you perform standardized screening for alcohol use during obstetric visits? (This does not include discussing alcohol use after a patient brings up the topic.)

All of the time

Most of the time 

Sometimes

Rarely

Never

Q8 How comfortable are you screening for alcohol use during obstetric visits?

Very comfortable

Somewhat comfortable

Not very comfortable

Not at all comfortable

Display the following question if you answered “All of the time”, “Most of the time”, “Sometimes”, or “Rarely” to Q7

Q9 How do you screen for alcohol use during obstetric visits? (Select up to three methods used most often.)

The patient fills out an intake questionnaire directly quantifying alcohol use.

The patient fills out an intake questionnaire using alcohol screening methods (CAGE, AUDIT-C).

An MA/LPN/RN directly quantifies alcohol use during intake.

An MA/LPN/RN uses screening methods (CAGE, AUDIT-C) during intake.

I directly quantify alcohol use during my interview.

I use alcohol screening methods (CAGE, AUDIT-C) during my interview.

Display the following question if you answered “Never” to Q7

Q10 What are the reasons you do not routinely screen for alcohol use during obstetric visits? (Select up to three of the most common reasons.)

There is not enough time during clinic visits.

Screening for at-risk or harmful alcohol use is outside the scope of OB/GYN and should be done in primary care.

I am worried it will interfere with patient/provider relationship.

I am worried it will lead to legal consequences for the patient.

I do not feel I have the ability to effectively screen for at-risk or  harmful alcohol use.

I do not feel I have the ability to effectively counsel patients about at-risk or harmful alcohol use.

I do not feel I have the ability to effectively refer patients with at-risk or harmful alcohol use for treatment.

Q11 Do you ever counsel pregnant patients, or patients hoping to become pregnant, on alcohol use during pregnancy?

Yes

No

Q12 Which of the following best describes what you recommend as the upper limit for a safe amount of alcohol in a pregnant patient?

I do not feel any alcohol use is safe during pregnancy

No more than 1 drink per week

No more than 1 drink per day, Up to 4 days a week

No more than 1 drink per day, Up to 7 days a week

No more than 2 drinks per day, Up to 4 days a week

No more than 2 drinks per day, Up to 7 days a week

I do not have a recommendation

Header 3 Identification of and Counseling for At-Risk or Harmful Alcohol Use
 

The following set of questions refers to both Obstetric and Gynecology patient visits.
 

Q13 How comfortable are you performing each of the following? (Very Comfortable, Somewhat Comfortable, Not Very Comfortable, Not at all Comfortable, Not very comfortable, Not at all comfortable)

Counseling patients on “healthy alcohol use” 

Identifying at-risk or harmful alcohol use

Identifying patients with alcohol use disorder ("alcoholism")

Discussing psychological risks for patients who drink heavily (for example, anxiety, depression, etc.)

Discussing reproductive health risks for patients who drink heavily (for example, high risk sexual behaviors, STIs, etc.)

Discussing medical risks for patients who drink heavily (for example, high blood pressure, liver disease, etc.)

Discussing risks to the fetus for pregnant people who drink heavily (for example, fetal alcohol spectrum disorders, etc.)

Header 4 Interventions for At-Risk or Harmful Alcohol Use The following set of questions refers to both Obstetric and Gynecology patient visits.

Q14 What actions have you taken when you have identified patients with at-risk or harmful alcohol use? (Select up to three most commonly used.)

Counseled patients on safe alcohol intake

Referred patients to social work for further evaluation

Referred patients to addiction specialist

Recommended patients discuss the behavior with their primary care provider 

Referred patients to gastroenterology or hepatology

Header 5 Evaluation for Alcohol-Associated Liver Disease The following set of questions refer to both Obstetric and Gynecology visits.

Q15 Have you ever ordered labs (hepatic function panel, etc.) in a patient who you suspected had alcohol-associated liver disease?

Yes

No

Q16 Have you ever ordered imaging (ultrasound, etc.) in a patient who you suspected had alcohol-associated liver disease?

Yes

No

Header 6 Experience with At-Risk or Harmful Alcohol Use and Alcohol-Associated Liver Disease The following set of questions refer to both Obstetric and Gynecology patient visits.

Q17 Have you ever taken care of a patient you knew had a history of at-risk or harmful alcohol use?

Yes

No

Q18 Have you ever taken care of a patient with alcohol-associated liver disease (i.e., “fatty liver” on imaging, elevated liver enzymes attributed to alcohol use)?

Yes

No

Q19 Have you ever taken care of a patient with significant alcohol-associated liver disease (cirrhosis, alcohol-associated hepatitis with jaundice)?

Yes

No

Q20 Have you ever taken care of a patient who has been listed for liver transplant for alcohol-associated liver disease?

Yes

No

Q21 Have you ever taken care of a patient who has died of alcohol-associated liver disease?

Yes

No

Header 7 Education for At-Risk or Harmful Alcohol Use and Alcohol-Associated Liver Disease
 

Q22 Do you feel you received adequate training on at-risk or harmful alcohol use in your residency or fellowship training?

Yes

No

Q23 Do you feel there are adequate resources to learn more about at-risk or harmful alcohol use geared towards OB/GYNs?

Yes

No

Q24 Do you feel there are adequate resources to learn more about alcohol-associated liver disease geared towards OB/GYNs?

Yes

No

Q25 Would you be interested in learning more about at-risk or harmful alcohol use and alcohol-associated liver disease?

Yes

No

Header 8 Practice Demographics

Q26 What is your primary practice affiliation?

University-Based Academic Setting

Non-University-Based Community/Private Practice

Veteran’s Affairs

Other (please describe): __________________________________________________

I am not currently performing any clinical care

Q27 How would you best describe the location of your practice?

Urban 

Suburban

Rural

Q28 What are the estimated percentages of inpatient versus outpatient care? (Total must add up to 100%)

Inpatient Care: _______

Outpatient Care: _______

Total: ________

Q29 What are the estimated percentages of Obstetric Care versus Gynecologic Care in outpatient visits? (Total must add up to 100%)

Obstetric Care: _______

Gynecologic Care: _______

Total: ________

Q30 What type(s) of insurance does your practice accept? (Select all that apply.)

Private Insurance/HMO

Medicaid

Uninsured/Self Pay

Other (please specify): __________________________________________________

Header 9 Provider Demographics

Q31 What is your age?

________________________________________________________________

Q32 What is your current gender identity?

Male

Female

Nonbinary

Prefer to self-describe: __________________________________________________

Q33 What race(s) do you consider yourself? (Select all that apply.)

American Indian or Alaskan Native

Asian

Black or African American

Native Hawaiian or Other Pacific Islander

White or Caucasian

Prefer to self-describe: __________________________________________________

Q34 Are you Hispanic or Latino?

Yes

No

Q35 What best describes your medical training background?

M.D. (Doctor of Medicine) followed by OB/GYN residency

D.O. (Doctor of Osteopathic Medicine) followed by OB/GYN residency

Physician Assistant

Certified Nurse Midwife

Nurse Practitioner

Other (please list) __________________________________________________

Q36 How many years has it been since you completed your obstetrics/gynecology training?

I am still in training (residency, fellowship)

0-5

6-10

11-15

More than 15

Q37 Do you perform any of the following “health maintenance” tasks if you feel they are indicated? (Yes/No)

Lipid screening

Diabetes screening

Colon cancer screening

Breast cancer screening

Screening for domestic violence

Screening for tobacco use

Screening for substance use

Screening for risky non-substance use behaviors (seatbelt use, guns in home, etc.)

Q38 In your opinion, how much of a problem is at-risk or harmful alcohol use in your community?

No problem at all

A small problem

A moderate problem

A big problem
