# Correction: Obstetrics and Gynecology Provider Experience Screening for Harmful Alcohol Use: An Unmet Need for Standardized Screening and Intervention

**DOI:** 10.7759/cureus.c268

**Published:** 2025-08-26

**Authors:** Mary J Thomson, Cresta Jones, Nicholas Lim

**Affiliations:** 1 Gastroenterology, Hepatology and Nutrition, University of Minnesota, Minneapolis, USA; 2 Obstetrics, Gynecology and Women's Health, University of Minnesota, Minneapolis, USA

The Ethics Statement and Conflict of Interest Disclosures section of this article has been corrected to indicate that this study did involve human subjects.

